# Radiocarbon dataset for the TRB central-place at Kałdus, Poland

**DOI:** 10.1016/j.dib.2024.110707

**Published:** 2024-07-05

**Authors:** Kamil Adamczak, Magdalena Kozicka, Łukasz Kowalski, Dominika Kofel, Wojciech Chudziak, Piotr Błędowski, Jacek Bojarski, Ryszard Kaźmierczak, Marcin Weinkauf

**Affiliations:** a Institute of Archaeology, Centre for Applied Archaeology, Nicolaus Copernicus University in Toruń, Szosa Bydgoska 44/48, 87-100 Toruń, Poland; b Institute of Archaeology, Nicolaus Copernicus University in Toruń, Szosa Bydgoska 44/48, 87-100 Toruń, Poland

**Keywords:** 14 C dating, Bayesian modelling, Late Neolithic, Funnel beaker culture, Baden culture, Central Europe

## Abstract

This dataset compiles radiocarbon dates received for botanical macroremains and animal bones from domestic and ritual pits and human graves unearthed during excavations at the archaeological site of Kałdus (Poland) that can be related to the Funnel Beaker culture (TRB). Prior to radiocarbon dating by accelerator mass spectrometry (AMS), plant macroremains were checked against diagnostic attributes of species identification by standard paleobotanical analysis. The dataset contains already published (*n* = 4) and new (*n* = 10) radiocarbon dates that were used to establish the absolute chronology of the TRB habitus at Kałdus and its diachronic spatial organization. This dataset serves as an archive for future studies focusing on the TRB settlement pattern and organization in the region of modern Poland. It also has a utility to be reused in archaeological and chronological research on the movement of copper metalwork and the gradual spread of human cremation rite in the region.

Specifications Table

**Table:** 

Subject	Archaeology
Specific subject area	AMS radiocarbon dating, Bayesian modelling
Type of data	Table, Figure Raw, Processed
Data collection	Radiocarbon dating: an Accelerator Mass Spectrometer (AMS) National Electrostatics Corporation (NEC) 1.5 SDH Compact Carbon Pelletron at Poznań ^14^C dating unit (Poznań Park of Science and Technology, Poland). Archaeobotanical analysis: for soil samples a manual bucket flotation system was used. The macroscopically visible plant remains from flotation and those extracted from potsherds and daub lumps were identified from morphological characters using a Delta Optical SZ-430B low-power stereomicroscope (magnification ×6.5–40).
Data source location	Kałdus (53°19′41,178″N 18°22′52,010″E), Chełmno commune, Poland
Data accessibility	Repository name: Mendeley Data Data identification number: 10.17632/tdxv8×7b7r.1 Direct URL to data: https://data.mendeley.com/datasets/tdxv8×7b7r
Related research article	

## 1 Value of the Data

• This dataset brings together all radiocarbon dates for the Late Neolithic (TRB) habitus at the site of Kałdus, which is one of the northernmost archaeological testimonies of the Baden culture uptake in Central Europe.
• The database includes two pits with one of the earliest copper metal depositions in Central Europe, which are rarely radiocarbon dated.
• This dataset offers the first direct radiocarbon evidence of a one-phase, large TRB settlement in the region.
• Archaeologists can use this dataset for the interregional research on the emergence and development of the Baden culture north of the Carpathians.
• The data have utility for refining the absolute chronology of the TRB settlement in the region.

## 2 Background

The problem of large sites of the Funnel Beaker (TRB) culture has long been recognized in Polish archaeology [[1], [2], [3]]. Although in some cases the TRB settlements of considerable extent have been brought to light by excavation (e.g. Bronocice, Ćmielów, Gródek Nadbużny, Mozgawa), their exposure represents only a small portion of the total area of a real settlement coverage. The delineation of a settlement area is usually determined in relation to the distribution of features and structures recorded at the site and their stratigraphic relations. This fact, combined with the shortage of radiocarbon data for the extensive TRB sites in Poland, has fueled the ongoing debate as to whether such archaeological records offer evidence of large settlements, encompassing an area of up to 30–50 ha, or represent a multi-phase settlement with diachronic spatial organization.

The original motivation behind compiling this dataset was to fill the gap in radiocarbon dating of the TRB settlement at Kałdus, north-central Poland, and provide an absolute chronology framework for investigating the spatial and temporal organization of the site. This dataset offers a starting point for creating an open access radiocarbon database for the site, which will be gradually updated as new radiocarbon dates become available. Presented information can be further explored and placed within the wider settlement context of the Late Neolithic in the region of modern Poland and beyond.

## 3 Data Description

**Fig. 1:**
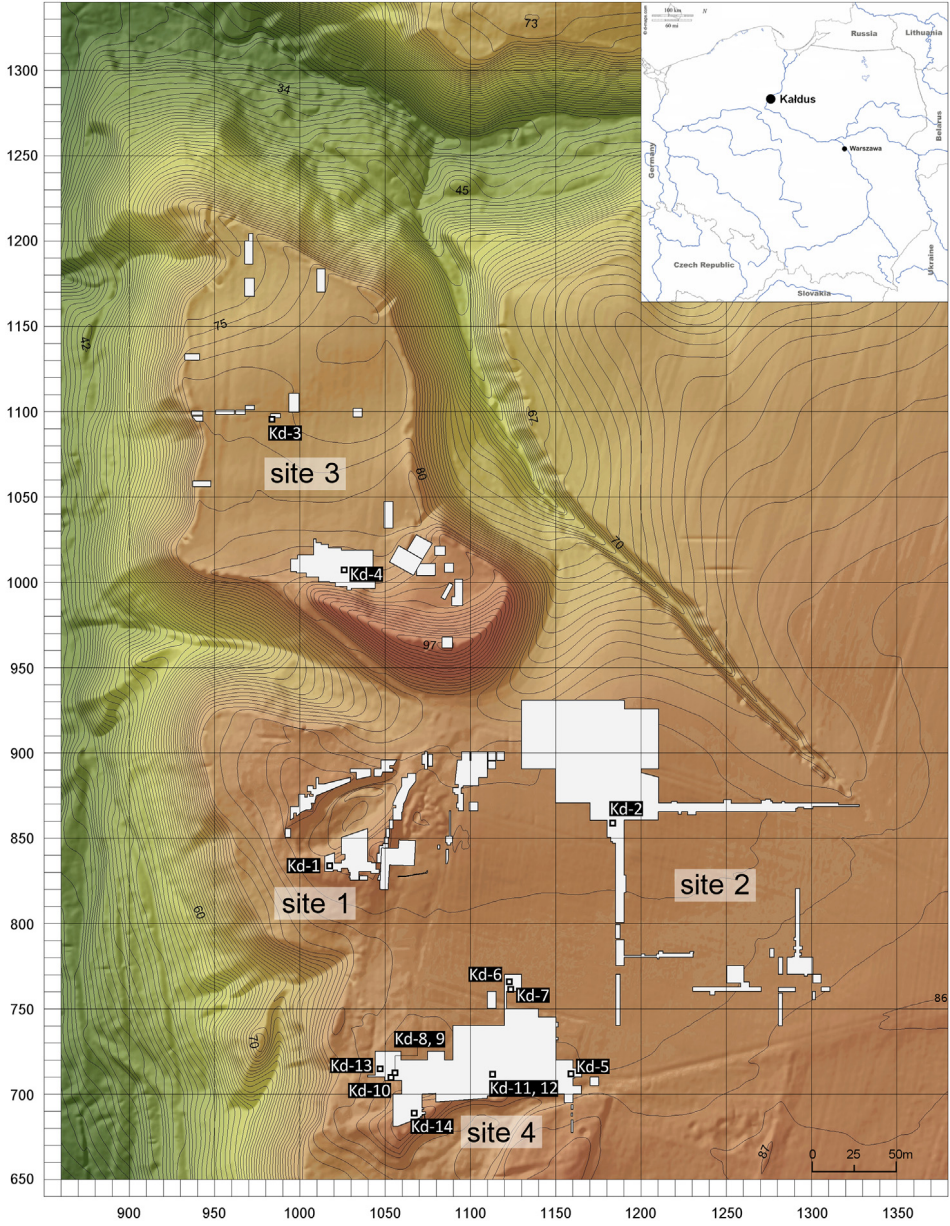
Plan of the archaeological site in Kałdus showing the location of features and layers covered by this dataset (plan site by M. Skrzatek; edited by Ł. Kowalski; map background: https://d-maps.com; see Table 1 for further details).

This dataset consists of 14 AMS radiocarbon dates of charged material and bulk collagen extracted from one soil sample (No. Kd-13), two fragments of daub, three potsherds, three pieces of unidentified charred material and five animal bones from the Late Neolithic habitus at the site of Kałdus, north-central Poland (Fig. 1). Two pits were radiocarbon dated twice (sample Nos. Kd-8 and 9, and Kd-11 and 12). Archived archaeological materials and documentation for the site were revisited in 2018. The sampled archaeological material was collected by the authors during excavations at the site between 1996 and 2016 [4], and analyzed in the Poznań Radiocarbon Laboratory (Poland).

All faunal and botanical remains were collected from domestic and ritual pits and human graves. These features can be linked to the TRB habitus at the site and dated by ceramic typology to as early as the mid-4th millennium BC [[5], [6], [7]].

Table 1 summarizes all AMS radiocarbon dates with their archaeological context, which includes site location, context of dated material and accompanying finds. For animal bones and botanical macroremains taxonomic identification is provided. The bibliographical references of the primary radiocarbon data are referenced in brackets on the column “^14^C Lab ID” in Table 1 and in extensive form in the References. The location of features and layers covered by this dataset is shown in Fig. 1.

**Table 1:** Summary of radiocarbon dates for the TRB habitus at Kałdus covered by this dataset, with their archaeological context and references of the primary data (in brackets). Sample ID corresponds to number shown on the map in Fig. 1. Two pits have two radiocarbon dates (sample Nos. Kd-8 and 9, and Kd-11 and 12).

Sample ID	Dated material	Species	Site-no.	Context	Accompanying finds	^14^C Lab ID	Age BP	Age BC	
								68.2 %	95.4 %
Kd-1	botanical macroremains	*Tricitum*	1	storage pit (?)	potsherds, daub, animal bones	Poz-101794	4690±30	3519–3377	3627–3371
Kd-2	animal bone	unspecified	2	ritual pit	Copper objects (ornament, hammer-axe, dagger), bone awl, amber disc, clay spindle whorl, flint retouched blade, two flint arrowheads, potsherds	Poz-95657 [6]	4640±40	3502–3363	3620–3350
Kd-3	botanical macroremains	*Tricitum*	3	storage pit	ceramic vessel, potsherds	Poz-128177	4520±35	3353–3106	3361–3099
Kd-4	botanical macroremains	*Tricitum*	3	house (?)	ceramic vessels, daub	Poz-102080	4590±50	3499–3128	3516–3103
Kd-5	charcoal	unspecified	4	grave	potsherds, daub, flint artefacts, animal bones	Poz-101796	4880±35	3695–3642	3759–3541
Kd-6	animal bone	unspecified	4	grave	ceramic vessels, potsherds, flint artefact, human bones (cremated), animal bones	Poz-127982	4500±35	3338–3104	3357–3041
Kd-7	botanical macroremains	*Tricitum*	4	grave	ceramic vessels, potsherds, flint artefact, grinding stone, animal bones	Poz-128178	4565±35	3371–3120	3492–3102
Kd-8	animal bone	*Sus scrofa f. domestica*	4	ritual pit	copper ornament, ceramic vessels, polishing stone, animal bones, potsherds	Poz-97720 [6]	4620±40	3498–3356	3520–3138
Kd-9	animal bone	*Sus scrofa f. domestica*	4	ritual pit	see “Kd-8”	Poz-97721 [6]	4640±40	3502–3363	3620–3350
Kd-10	animal bone	*Ovis aries/Capra hircus*	4	ritual pit (?)	flint axe, bone perforator, animal bones, potsherds	Poz-101200 [7]	4640±40	3502–3363	3620–3350
Kd-11	charcoal	unspecified	4	storage pit	ceramic vessel, potsherds, millstone	Poz-102079	4500±50	3338–3103	3362–3027
Kd-12	botanical macroremains	*Tricitum*	4	storage pit	see “Kd-11”	Poz-128180	4540±50	3366–3105	3489–3036
Kd-13	botanical macroremains	*Hordeum vulgare*	4	trash pit	potsherds, animal bones	Poz-87412	4680±30	3516–3376	3623–3370
Kd-14	charcoal	unspecified	4	trash pit	ceramic vessel, potsherds, daub, flint artefacts, stone artefact, animal bones	Poz-101795	4735±30	3631–3384	3635–3378

**Fig. 2:**
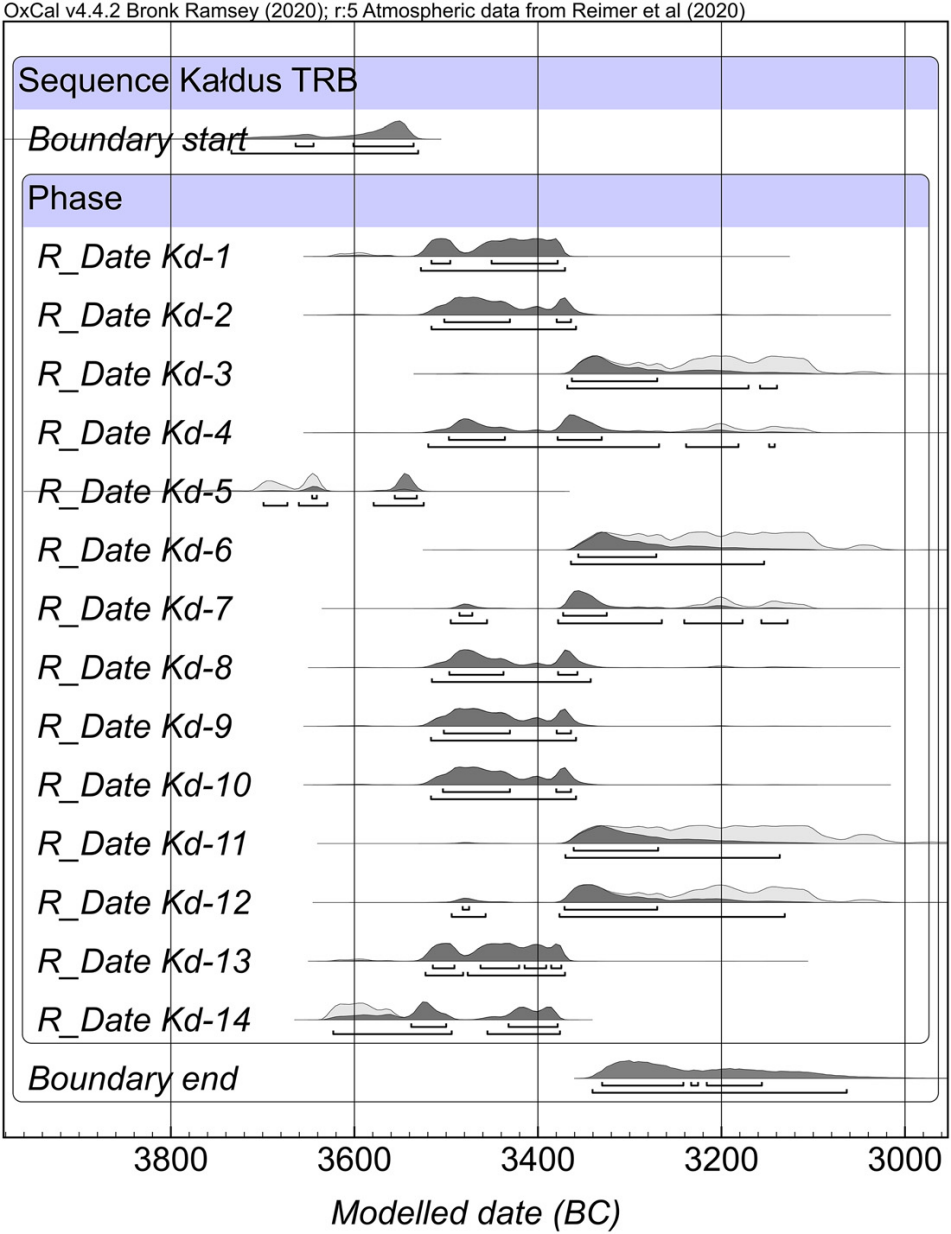
One-phase model for the TRB habitus at Kałdus covered by this dataset (OxCal v4.4.2; see Table 2 for further details).

**Fig. 3:**
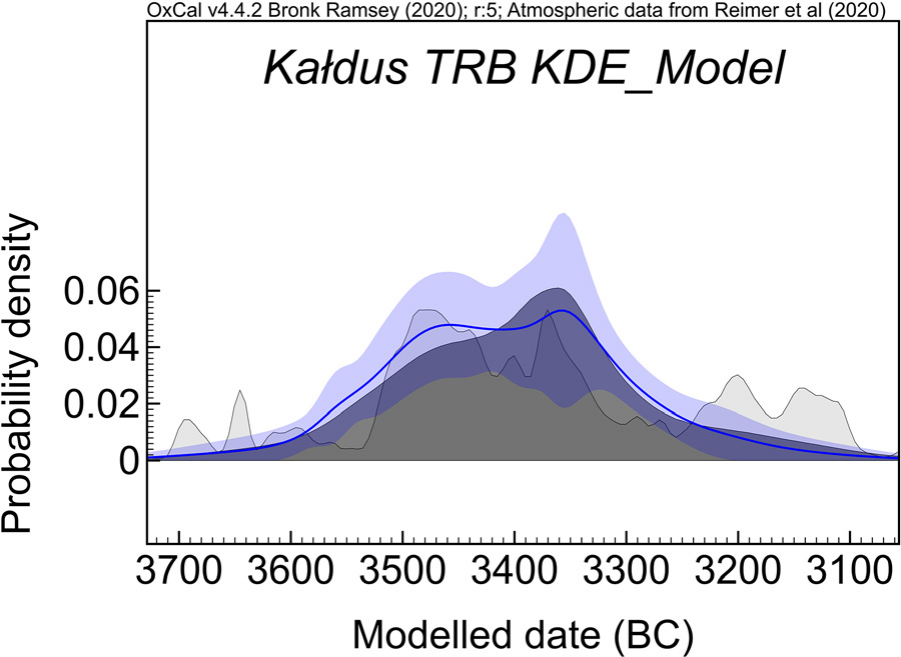
The KDE model for the TRB habitus at Kałdus covered by this dataset (OxCal v4.4.2; A_overall_=88.9; A_model_=88.2). The dark grey is the KDE estimated distribution of the events at the site. The light grey is the sum of the calibrated date distribution. The blue line and band provide *a* ± 1σ estimate of the KDE model uncertainty.

Summary of a single-phase model for the TRB habitus at the site is given in Table 2 and Fig. 2. The Kernel density model (KDE) distribution of radiocarbon dates is shown in Fig. 3.

**Table 2:** Summary of a single-phase model for the TRB habitus at Kałdus covered by this dataset (A_overall_=85.1; A_model_=84) (OxCal v4.4.2).

Command	Sample ID	Unmodelled age BC		Modelled age BC		Agreement index
		68.2 %	95.4 %	68.2 %	95.4 %	
Boundary start	…	…	…	3663–3536	3726–3531	…
	Kd-1	3517–3379	3605–3370	3517–3379	3528–3371	102.9
	Kd-2	3504–3365	3521–3356	3504–3365	3517–3360	101
	Kd-3	3353–3106	3361–3099	3364–3282	3369–3141	96.3
	Kd-4	3497–3126	3516–3102	3497–3332	3520–3183	113.8
	Kd-5	3704–3636	3765–3536	3647–3532	3697–3525	41.2
	Kd-6	3338–3104	3357–3041	3357–3276	3365–3158	98
	Kd-7	3371–3120	3492–3102	3486–3326	3496–3130	119.9
	Kd-8	3498–3357	3522–3193	3497–3358	3516–3344	102.9
	Kd-9	3504–3365	3521–3365	3504–3365	3517–3360	101.1
	Kd-10	3504–3365	3521–3365	3503–3365	3517–3360	101.2
	Kd-11	3339–3103	3364–3026	3362–3275	3371–3139	101.4
	Kd-12	3366–3105	3498–3036	3484–3275	3495–3132	99.9
	Kd-13	3516–3375	3526–3369	3515–3376	3523–3371	101.4
	Kd-14	3626–3383	3632–3377	3539–3379	3622–3377	91.9
Boundary end	…	…	…	3334–3170	3340–3071	…

## 4 Experimental Design, Materials and Methods

### 4.1 Study area

The multi-period archaeological site at Kałdus is located in north-central Poland, on the border of the Lower Vistula Valley and the Chełmno-Dobrzyń Lakeland, ca. 3 km south of Chełmno (53°19′41,178″N 18°22′52,010″E). The Kałdus site covers a total area of ca. 15 ha and stretches over four conventional sites (Fig. 1). The site was first excavated by Lissauer in 1877 and fieldworks were continued by Polish and German archaeologists in the late 19th c. and at the beginning of the 20th c. Much more archaeological record was gained from the site after World War II. In 1996, an extensive research program was run in Kałdus by the Institute of Archaeology, NCU in Toruń, encompassing the entire site complex and focusing on the relics of the Early Middle Ages [4].

To date, approximately 15 % of the total site area (over 2 ha) has been excavated, exposing archaeological structures and objects that signify domestic and ritual activities at the site during an extended timescale spanning the Late Neolithic to the Early Middle Ages. The bottom layers recorded in Kałdus can be dated by ceramic typology to as early as the mid-4th millennium BC and linked to the TRB culture. The TRB's activity has been exposed over the entire area of the site and can be attested to by cremation graves, metal hoards and numerous storage and ritual pits, which yielded a substantial portion of ceramics and other archaeological material. The Neolithic habitus in Kałdus was succeeded by the Early Bronze Age newcomers of the Iwno culture (ca. 2100–1700 BC), and later by the Lusatian culture of the Early Iron Age (ca. 750–450 BC). The uppermost stratum of the site was formed between the 11th and 13th c. AD, when Kałdus became the political center (*sedes regni principalis*) for the first rulers of Piast Poland [[4], [5], [6], [7]].

### 4.2 Archaeobotanical analysis

The soil samples, potsherds and daub fragments were analyzed to extract material for radiocarbon dating. Soil sampling followed a combination of a systematic and judgement sampling strategies [8] and used a manual bucket flotation system. Each soil sample was dispersed in water and then gently stirred to release the botanical remains. Thereafter, the watery solution from the upper part of the bucket was poured through two sieves (0.5 mm and 0.25 mm mesh size). The next step was to pour fresh water onto the soil remains at the bottom of the bucket and the operation was repeated until no more soil was left. Sieves retained both the heavy and the light residues after silts and other particles smaller than 0.25 mm were rinsed through. Residues were dried and the heavy elements were separated from the lighter ones. After that, the residues were sorted under a Delta Optical SZ-430B low-power stereomicroscope (magnification ×6.5–40). The macroscopically visible plant remains were picked from the different sieved residues and identified from morphological characters. The macrofossil identifications were checked against the botanical literature [9,10] and compared with the modern reference collection, following standard nomenclature [[9], [10], [11]]. The potsherds and daub fragments were initially measured and then hand cleaned using soft brush and rubber dust blower. Observations of imprints were made with the use of a low-power stereomicroscope at low magnifications (max ×20), after which the ceramic objects were crushed for more negatives and charred plant fragments. Taxonomical identifications followed the botanical literature and were compared with the modern reference collection.

### 4.3 Radiocarbon dating

Selected botanical macroremains, charcoal and animal bones were analyzed in the Poznań Radiocarbon Laboratory (Poland). Preparation of the samples and dating by Acceleration Mass Spectrometry (AMS) was performed according to procedures described by Goslar [12]. All dates were calibrated in OxCal v4.4.2 [13] against the IntCal20 calibration curve [14]. The resulting endpoints were not rounded and the radiocarbon ages are reported as 68.2 % and 95.4 % confidence intervals. The data on the quality of the bone collagen can be found in the .xlsx file shared in the repository.

### 4.4 Bayesian modelling

KDE and one-phase Bayesian start/end date modelling [[15], [16], [17]] were performed in OxCal v4.4.2 [13] using the IntCal20 calibration curve [14] to summarize the radiocarbon dates and obtain a refinement of the TRB habitus at Kałdus.

### 4.5 Preliminary data analysis

The Bayesian modelling refined the radiocarbon dataset for Kałdus by providing a potential start date for the TRB habitus at 3663–3536 cal BC and suggests that the end boundary was 3334–3170 cal BC, both at 68.3 % (Table 2 and Fig. 2). According to the KDE model (Fig. 3), the events recorded at the site that are covered by this dataset (Table 1) are tightly clustered in a relatively short time period of approximately 200 years ranging between 3520 cal BC and 3320 cal BC. The Bayesian modelling suggests a continuous occupation of Kałdus by the TRB people, which extended over ca. 15 ha of the site (Fig. 1) and 8–10 generations.

## Limitations

At the moment, only the site 4 has representative coverage of radiocarbon dates. As the Kałdus site complex has been excavated with a particular focus on the Early Middle Ages activity, no systematic programme of archaeobotanical sampling was carried out for features and structures that have been assigned to the Late Neolithic. The radiocarbon dating of TRB pottery from the site revealed that the ceramic paste used for their production contains different carbon sources, of different radiocarbon ages. Furthermore, the obtained date ranges overlap and enter the plateau region of the calibration curve beginning around 3350 cal BC. Radiocarbon database for the TRB habitus at Kałdus will be gradually updated as new radiocarbon dates become available.

## Ethics Statement

This work does not involve any modern human subjects, animal experiments, or any data collected from social media platform.

## CRediT authorship contribution statement

**Kamil Adamczak:** Writing – original draft, Funding acquisition. **Magdalena Kozicka:** Data curation, Methodology, Software, Writing – original draft, Visualization, Funding acquisition. **Łukasz Kowalski:** Conceptualization, Writing – original draft, Writing – review & editing, Visualization, Supervision. **Dominika Kofel:** Writing – original draft, Investigation. **Wojciech Chudziak:** Resources, Funding acquisition. **Piotr Błędowski:** Resources. **Jacek Bojarski:** Resources. **Ryszard Kaźmierczak:** Resources. **Marcin Weinkauf:** Resources.

## Data Availability

14C DATABASE for the TRB site of Kałdus, PL (Original data) (Mendeley Data)
